# Eosinophilic Granulomatosis with Polyangiitis (EGPA): A case report with atypical presentation

**DOI:** 10.12669/pjms.39.1.6436

**Published:** 2023

**Authors:** Khalid Mahmood, Nauman Ismat Butt, Fahmina Ashfaq, Sabeen Aftab

**Affiliations:** 1 Khalid Mahmood, MBBS, FCPS. Assistant Professor, Department of Medicine & Allied, Azra Naheed Medial College, Superior University, Lahore, Pakistan; 2 Nauman Ismat Butt, MBBS, FCPS. Assistant Professor, Department of Medicine & Allied, Azra Naheed Medial College, Superior University, Lahore, Pakistan; 3 Fahmina Ashfaq, MBBS, MRCP, MCCEE. Assistant Professor, Department of Medicine & Allied, Azra Naheed Medial College, Superior University, Lahore, Pakistan; 4 Sabeen Aftab, MBBS, FCPS. Assistant Professor, Department of Medicine & Allied, Azra Naheed Medial College, Superior University, Lahore, Pakistan

**Keywords:** Eosinophilic Granulomatosis with Polyangiitis, ANCA-associated Vasculitis, Eosinophilia, Pulmonary Vasculitis

## Abstract

A 72-year-old asthmatic gentleman with a history of recurrent sinusitis and chronic bronchitis presented with shortness of breath and progressively worsening hypoxemic respiratory failure. His CT chest demonstrated airspace disease bilaterally with ground-glass opacifications. He had peripheral eosinophilia with raised inflammatory markers but negative work up of infection. On further investigation, ANA was positive, titer 1:160, speckled pattern and both pANCA and cANCA were present. The patient was diagnosed with Eosinophilic Granulomatosis with Polyangiitis (EGPA) and started on intravenous steroids and cyclophosphamide. A rare multi-organ vasculitis, EGPA is hallmarked by asthma, sinusitis and eosinophilia. In initial stages vasculitic involvement is not usually seen thereby making EGPA a diagnostic challenge.

## INTRODUCTION

Eosinophilic Granulomatosis with Polyangiitis (EGPA), a rare multisystem ANCA-associated vasculitis, is defined by eosinophil-predominant, granulomatous inflammation with necrosis usually affecting the respiratory passages, necrotizing vasculitis involving medium-to-small sized blood vessels, asthma and eosinophilia.^1^ It typically affects young adults aged 40 to 60 years and shows no sex difference.^2^ It usually develops in three sequential phases.^2^,^3^ the allergic phase characterized by asthma, allergic sinusitis and rhinitis; the eosinophilic phase demonstrated by eosinophilic infiltrations in organs e.g. pulmonary, gastrointestinal and cardiac systems; the vasculitic phase distinguished by purpura, mononeuritis multiplex and constitutional symptoms like fever, lethargy, fatigue and weight loss. Anti-Neutrophil Cytoplasmic Antibodies (especially p-ANCA, anti-myeloperoxidase) are seen in up to 60% patients.^3^

## CASE PRESENTATION

**Fig.1:**
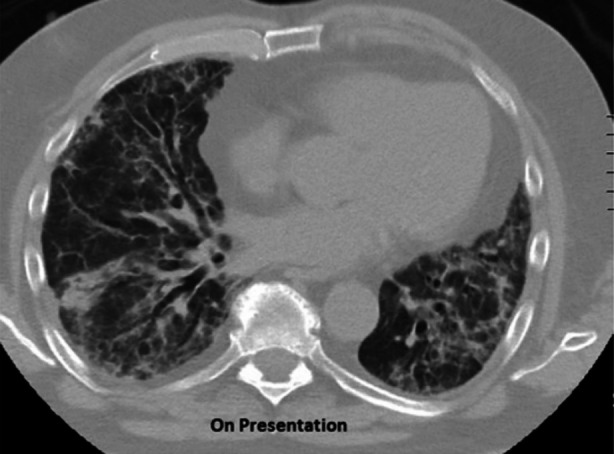
HRCT chest on presentation.

**Fig.2:**
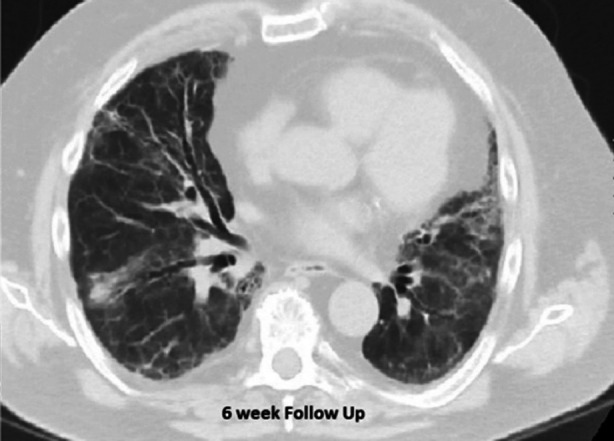
HRCT chest at 6 weeks follow up.

We present the case of a 72-year-old asthmatic gentleman with a history of recurrent sinusitis and chronic bronchitis. He was admitted in the Intensive Care Unit with sudden onset shortness of breath and progressively worsening hypoxemic respiratory failure and was commenced on high flow oxygen (60LPM; FiO2 90%-100%) via nasal cannula. On examination, bilateral chest crackles were noted with no pedal edema. X-ray followed by CT chest was done on presentation to show airway disease bilaterally with ground-glass opacifications as shown in Fig.1. His CBC revealed peripheral eosinophilia (23.8%) with normal Hemoglobin level and platelet count. On further investigation, he had raised ESR and CRP but serum procalcitonin, renal function, serum electrolytes and BNP were normal. His SARS-CoV-2 PCR was negative twice, done 24 hours apart. Autoimmune serology was sent and empirical antibiotics started. The patient did not consent for bronchoscopy. Workup for infections including blood and sputum culture was negative. Autoimmune serology demonstrated raised ANA with titer 1:160 and speckled pattern. He had raised p-ANCA with anti-MPO antibody titer 49.4 and raised c-ANCA with anti- anti-PR3 antibody titer 18.8. The patient was diagnosed with Eosinophilic Granulomatosis with Polyangiitis (EGPA) based on history of asthma, sinusitis, eosinophilia, pulmonary vasculitis, positive p-ANCA and c-ANCA. Intravenous methylprednisolone was started along with intravenous cyclophosphamide after which his oxygen need showed improvement. Subsequently, he was discharged on oral steroids with a plan to infuse cyclophosphamide once a month for induction of remission. After six weeks, follow-up CT chest revealed marked improvement as shown in Fig.2.

## DISCUSSION

The clinical features of EGPA are variable with some manifestations preceding others leading to delay in diagnosis of EGPA to later stages. The clinical features of EGPA can be divided into two presentations: the ANCA positive *vasculitic picture* resulting from medium-to-small sized blood vessels involvement showing features like purpuric rash, glomerulonephritis and mononeuritis multiplex; the ANCA negative *eosinophilic* pattern in which eosinophilic infiltration leads to organ damage characterized by pulmonary infiltrates and cardiomyopathy.^4^ However these subsets are not well-demarcated and overlapping features of both are seen commonly. On investigation, marked hypereosinophilia is the commonest finding in EGPA and increased ESR and CRP are also frequently seen.^5^ ANCA are seen in up to 60% patients with p-ANCA being the frequent pattern having myeloperoxidase specificity.^3^ The American College of Rheumatology (ACR) criteria encorporates asthma, mononeuritis multiplex, peripheral eosinophilia >10%, fleeting pulmonary infiltrates on radiography, abnormality of paranasal sinus, and biopsy demonstrating extravascular eosinophils.^6^ If four of these six criteria are a present, diagnosis of EGPA can be made with 99.7% specificity.^6^ Our patient had three of these criteria. BAL and biopsy were not done due to lack of consent by the patient but his autoimmune serology was atypical demonstrating both p-ANCA and c-ANCA in high titers.

Treatment of EGPA is a matter of debate because large-scale randomized controlled trials are lacking. For induction of remission, medical therapy incorporates immunosuppressive therapy with systemic steroids and cyclophosphamide.^7^ Rituximab has been shown to be an alternative approach in particular in cases where there is frequent recurrence or fear of cyclophosphamide toxicity.^8^ Side effects of cyclophosphamide include infertility, bone marrow suppression, increased infection risk, gastrointestinal upset (nausea, vomiting, anorexia) and an increase in malignancy risk.^7^ Additionally in life-threatening hemoptysis and deteriorating renal function, ventilator support, plasma exchange or pharesis and hemodialysis have been used in spite of immune-suppression. For maintenance of EGPA remission, azathioprine or methotrexate are generally used.^9^ Azathioprine and methotrexate require routine monitoring of blood counts and liver function. The five factors score (FFS) is used clinically with score of >1 associated with poor prognosis.^10^ FFS assigns one point to each of the following: neurological involvement, gastrointestinal involvement, cardiac involvement, proteinuria greater than 1 g/24 h and serum creatinine more than 141 μmol/L.^10^

## CONCLUSION

Eosinophilic Granulomatosis with Polyangiitis may affect various systems and can lead to potentially life-threatening complications. Timely diagnosis with early commencement of immune-suppressive therapy aids in inducing and maintaining remission.

### Authors’ Contribution:

**NIB and KM:** Conceived and designed were involved in manuscript writing

**FA and SA** did the initial literature search.

**NIB and FA** did the data collection, and patient assessment..

**SA and FA** did the final critical review and corrections.
